# Characterization and phylogenetic analysis of the complete mitochondrial genome of a basidiomycetous yeast *Cystobasidium* sp. (Cystobasidiales: Cystobasidiaceae)

**DOI:** 10.1080/23802359.2020.1777910

**Published:** 2020-06-16

**Authors:** Qiaofeng Liu, Xin Wang

**Affiliations:** a Department of Pathology and Pathophysiology, Chengdu Medical College, Chengdu, China; b Department of Pathogenic Biology, Chengdu Medical College, Chengdu, China

**Keywords:** *Cystobasidium*, basidiomycetous yeast, mitochondrial genome, phylogenetic analysis

## Abstract

In the present study, the complete mitochondrial genome of a basidiomycetous yeast *Cystobasidium* sp. was assembled and obtained. The mitochondrial genome of *Cystobasidium* sp. contains 16 protein-coding genes, 2 ribosomal RNA genes (rRNA), and 24 transfer RNA (tRNA) genes. The complete mitogenome of *Cystobasidium* sp. has a total length of 24,914 bp, with the base composition as follows: A (30.82), T (32.88%), C (18.37%) and G (17.93%). The *Cystobasidium* sp. mitogenome exhibited a close relationship with the mitogenome of *Microbotryum cf. violaceum*, *M. lychnidis-dioicae*, and *Rhodotorula mucilaginosa*.

The genus *Cystobasidium* belongs to Cystobasidiaceae, Cystobasidiales, which was first emended by Yurkov et al. (2015) to accommodate a group of closely related asexual species. *Cystobasidium* species is often pink pigmented, with ovoidal to elongate cells and polar budding (Turchetti et al. 2018). Species of this genus were reported to have biotransformation capacities (Vyas and Chhabra 2017; Tanimura et al. 2018). So far, about 16 species have been described in this genus (Chang et al. 2019). The complete mitochondrial genome of *Cystobasidium* sp. will promote the understanding of the phylogeny and evolution of the basidiomycetous yeast.

The specimen (*Cystobasidium* sp.) was isolated from a water sample collected in Chengdu, Sichuan, China (103.51E; 30.42N). The specimen was stored in Culture Collection Center of Chengdu Medical College (No. Cys03). The total genomic DNA of *Cystobasidium* sp. was extracted using Fungal DNA Kit D3390-00 (Omega Bio-Tek, Norcross, GA). Extracted DNA was purified using a Gel Extraction Kit (Omega Bio-Tek, Norcross, GA), and then stored in the Chengdu Medical College (No. DNA_ Cys03). Sequencing libraries were constructed with purified genomic DNA using the NEBNext^®^ Ultra™ II DNA Library Prep Kit (NEB, Beijing, China). Whole genomic sequencing (WGS) was performed by the Illumina HiSeq 2500 Platform (Illumina, San Diego, CA). The complete mitogenome was *de novo* assembled as implemented by SPAdes 3.9.0 (Bankevich et al. 2012). Gaps among contigs were filled by using MITObim V1.9 (Hahn et al. 2013). The obtained mitogenome was annotated according to the methods described by Li, Chen, et al. (2018), Li, Liao, et al. (2018), Li, Wang, et al. (2018).

The circular mitogenome of *Cystobasidium* sp. is 24,914 bp in size. The complete mitogenome contains 16 protein-coding genes, 2 ribosomal RNA genes (*rns* and *rnl*), and 24 transfer RNA (tRNA) genes. The base composition of the genome is as follows: A (30.82), T (32.88%), C (18.37%) and G (17.93%).

To investigate the phylogenetic status of *Cystobasidium* sp., we constructed phylogenetic trees of 18 species. Phylogenetic tree was constructed using Bayesian analysis (BI) method based on the combined 14 core protein-coding genes according to Li, Wang, Jin, Chen, Xiong, Li, Liu, et al. (2019), Li, Wang, Jin, Chen, Xiong, Li, Zhao, et al. (2019), Li, Yang, et al. (2020). As shown in the phylogenetic tree (Figure 1), the taxonomic status of the *Cystobasidium* sp. mitogenome exhibited a close relationship with the mitogenomes of *Microbotryum cf. violaceum* (KC285587), *M. lychnidis-dioicae* (KC285586), and *Rhodotorula mucilaginosa* (Gan et al. 2017).

**Figure 1.**
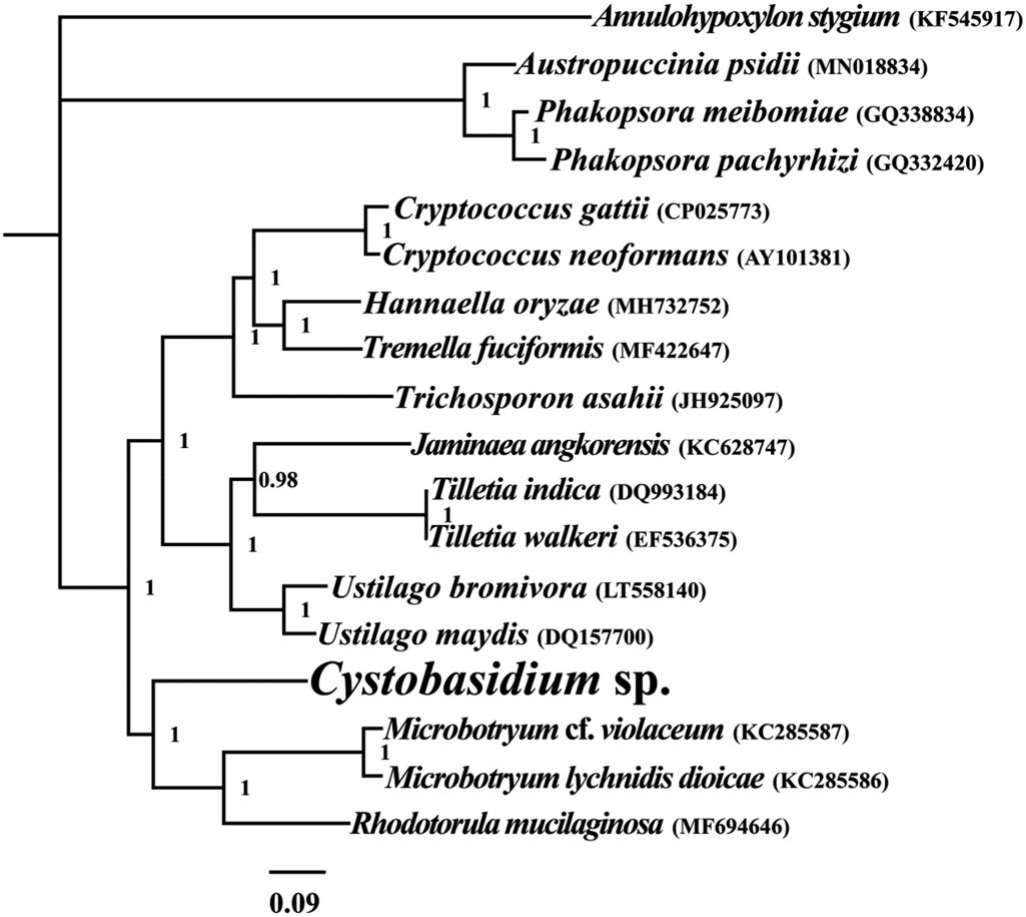
Bayesian phylogenetic analysis of 18 species based on the combined 14 core protein-coding genes. Accession numbers of mitochondrial sequences used in the phylogenetic analysis are listed in brackets after species.

## Data Availability

This mitogenome of *Cystobasidium* sp. was submitted to GenBank under the accession of MT366950 (https://www.ncbi.nlm.nih.gov/nuccore/MT366950).
